# Supplementary material for: Quantitative optical assessment of photonic and electronic properties in halide perovskite
Source: Nat Commun. 2019 Apr 8;10:1586. doi: 10.1038/s41467-019-09527-w (PMC6453959; doi:10.1038/s41467-019-09527-w)
Supplement: Supplementary file 1 — Supplementary Information [file 41467_2019_9527_MOESM1_ESM.pdf]

Supplementary Information

Quantitative optical assessment of photonic and electronic properties in halide perovskite

Adrien Bercegol[1,2], Daniel Ory[1,2], Daniel Suchet[2,3], Stefania Cacovich[2], Olivier Fournier[1,2], Jean Rousset[1,2] Laurent Lombez[2,3*]

[1] *EDF R&D, 30 RD 128, 91120 PALAISEAU, France*

[2] *IPVF, Institut Photovoltaïque d'Ile-de-France, 30 RD 128, 91120 PALAISEAU, France*

[3] *CNRS, Ecole Polytechnique, Institut Photovoltaïque d'Ile-de-France UMR 9006, 30 RD 128, 91120 PALAISEAU, France*

**Corresponding author : laurent.lombez@cnrs.fr*

Supplementary Note 1 General considerations for deriving the recycled contribution g_{rec}

g_{rec} is introduced in the article (equation (3)) to account for the generation due to photon recycling. In this section, we want to obtain analytical expressions allowing its calculation for any charge carrier distribution. (r, θ, z) coordinates will refer to the frame centered according to the pulsed point illumination at r=0. General considerations about photon emission inside an excited semi-conductor are first presented. Then, the photon flux is calculated for a distribution expressed in cylindrical coordinates around the regeneration point in two specific cases. The first one applies for calculation of $g_{\mathrm{corr}}=R_{\mathrm{eh}}/R_{\mathrm{eh}}^*$ at short distances, and derive its mean value used in the fit of electronic parameters. The second one applies at long distances, compared to the thickness of the film. It allows to fit the tail of the PL profiles.

In a first approach, we derive $g_{\mathrm{rec}}(\mathrm{O},t)$, where O is the origin of a frame with cylindrical coordinates, at the bottom of the perovskite layer, with absorption coefficient α (see Supplementary Figure 10), thickness z_0 (600 nm from[1]) , and refractive index n_{opt}. In the infinitesimal volume dV around X ($dV = dr \times rd\theta \times dz$), the excited charge carrier concentration $n(X)$ induces a generation of photons, the so-called photoluminescence[2]. This emission is isotropic and its photonic density n_γ per solid angle is proportional to its absorptivity $a(E)$, but also dependent on the local quasi-Fermi level splitting $\Delta\mu$:

$$n_\gamma(X, E, \Omega) = \frac{n_{\mathrm{opt}}^3}{4\pi\bar{h}^3\pi^2c^3}\frac{a(E)E^2}{\exp(\frac{E-\Delta\mu(X)}{k_{\mathrm{B}}T})-1} \approx \frac{K_{\mathrm{p}}n^2(X)}{n_0p_0}a(E)E^2\exp\left(\frac{-E}{k_{\mathrm{B}}T}\right) \quad (1)$$

The photon flux going out of a small surface dS_{em} (drawn on dV), in a direction defined by the solid angle $d\Omega_{\mathrm{em}}$ making an angle Ψ with the normal to dS_{em} would be expressed as :

$$j_\gamma = n_\gamma(X, E, \Omega)d\Omega_{\mathrm{em}}\cos(\Psi)dS_{\mathrm{em}}\frac{c}{n_{\mathrm{opt}}} \quad (2)$$

Note that we abbreviated all the physical constants into a single parameter K_{p}. Also, the ≈ sign refers to approximation of the Fermi-Dirac statistics with an exponential term $\exp\left(\frac{-E}{k_{\mathrm{B}}T}\right)$. We also converted the $\exp\left(-\frac{\Delta\mu}{k_{\mathrm{B}}T}\right)$ term into $\frac{n^2(X)}{n_0 p_0}$. $n_0 p_0$ can be determined as with the intrinsic radiative coefficient R_{eh} with[3]:

$$\int_E K_{\mathrm{p}} \alpha(E) E^2 \exp\left(\frac{-E}{k_{\mathrm{B}}T}\right) dE = \frac{R_{\mathrm{eh}} n_0 p_0}{4\pi} \quad (3)$$

Based on the measured absorption coefficient $\alpha(E)$ and fitted R_{eh} value, we obtain $n_0 p_0 = 4\text{x}10^{14}$ cm^{-3}. Now, we will distinguish two cases. The first one (Supplementary Note 2) refers to the case where $r \approx z_0$, when multiple reflexions of the PL on front and back interface can be neglected in a first approach. In fact, main part of the recycling comes from direct (in-plane) trajectories of the photon. The second one (Supplementary Note 3) refers to $r \gg z_0$, when total internal reflexions at front and back interfaces significantly enhance the intensity of the propagated flux. Indirect trajectories will have to be considered then.

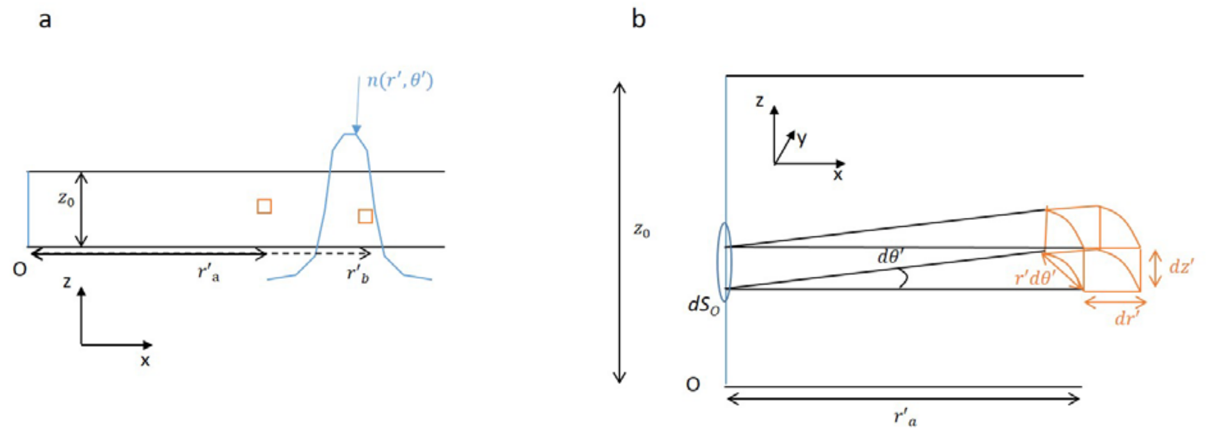

Supplementary Figure 1 Sketch of the elementary volume dV (orange box here above) considered in Supplementary Equations (5-6), drawn in a secondary frame. It is varied in the whole semi-conductor slab in Supplementary Equation (7). (a) lateral view of the semi-conductor slab with thickness $z_{0,}$ where the origin of the frame is distinct from the center of charge carrier distribution (blue line). (b) 3D sketch of the elementary volume.

Supplementary Note 2 Deriving g_{rec} at short range (r<z0 or r≈z0)

We start back from Supplementary Equation (2) that we adapt to derive the photonic flux $j_{\gamma 1}$ emitted towards the origin of a secondary frame (r', θ', z). This origin might be distinct from the center of charge carrier distribution as sketched in Supplementary Figure 1-a, where two elementary volumes at r'_a and $r'_b > r'_a$ are represented in such a secondary frame. Supplementary Figure 1-b displays a 3D view of the elementary volume, along with geometrical parameters allowing us to write $dS_{\mathrm{em}} = r' d\theta' dz$, and $d\Omega_{\mathrm{em}} = dS_O / r'^2$. The role of the angle Ψ in Supplementary Equation (2) is taken over by an in-depth integration from 0 to z_0. We integrate the flux $j_{\gamma 1}$ through an elementary surface dS_O placed at O ($r'=0$) to obtain the photon density $dn_{\gamma 1}$ at O. An attenuation term is added and corresponds to Beer-Lambert theory. It reads:

$$dn_{\gamma,1}(r', E) = \frac{j_{\gamma 1} \cdot n_{\mathrm{opt}}}{dS_O \cdot c} \exp(-\alpha(E) r')\, dE \quad (4)$$

Eventually, the photon density at the origin propagated from dV, can be written as below, provided that “$dS_O \cdot c$” factors get simplified:

$$dn_{\gamma,1}(r', \theta', E) = \frac{n_\gamma(r', \theta', E, \Omega)}{r'^2} \exp(-\alpha(E) r')\; r' d\theta' dz dE \quad (5)$$

In the next step, we replace $n_\gamma(r', E, \Omega)$, knowing that the absorptivity (and hence emissivity) of dV is $\alpha(E) dr'$.

$$dn_{\gamma,1}(r', \theta', E) = \frac{K_p \cdot n^2(r', \theta')}{n_0 \cdot p_0} \alpha(E) E^2 \exp\left(-\frac{E}{k_B T}\right) \frac{\exp(-\alpha(E) r')}{r'}\; dr' d\theta' dz dE \quad (6)$$

The integration over the whole volume remains. This formula remains valid if the charge carrier concentration is near the origin. Hence, the whole volume refers to r’ values comprised between 0 and some multiples of z_0 at most. Otherwise one should consider the second part of this calculation (Supplementary Note 3). It yields:

$$g_{\mathrm{rec}}(r) = \iiint_{(r', \theta', z')\text{frame centered in } (r,0,0)} \frac{K_p n^2(r', \theta')}{n_0 p_0} \ldots$$

$$\ldots \int_E \alpha^2(E) E^2 \exp\left(-\frac{E}{k_B T}\right) \frac{\exp(-\alpha(E) r')}{r'} dE\; dr' d\theta' dz' \quad (7)$$

The approximation of considering the PL as monochromatic with a single absorption coefficient α_{PR} is often seen in publications.

$$\alpha_{\text{PR}} = \frac{\int \alpha(E)E^2 \exp\left(-\frac{E}{k_\text{B}T}\right) dE}{\int E^2 \exp\left(-\frac{E}{k_\text{B}T}\right) dE} \quad (8)$$

It allows to separate spatial and energetic integrals as in Supplementary Equation (9) below, and we will comment on its validity.

$$\widetilde{g_{\text{rec}}}(r) = \iiint_{(r',\theta',z')\text{frame centered in }(r,0,0)} \frac{K_\text{p} n^2(r',\theta')}{n_0 p_0} \alpha_{PR} \frac{\exp(-\alpha_{\text{PR}} r')}{r\prime} \, dr' d\theta' dz' \ldots$$

$$\ldots \int_E \alpha(E)E^2 \exp\left(-\frac{E}{k_\text{B}T}\right) dE \quad (9)$$

This is equivalent to:

$$g_{\text{rec}}(r) = \iiint_{(r',\theta',z')\text{frame centered in }(r,0,0)} \frac{R_{\text{eh}} n^2(r',\theta')\alpha_{\text{PR}}}{4\pi} \frac{\exp(-\alpha_{\text{PR}} r')}{r\prime} \, dr' d\theta' dz' (10)$$

We applied this calculation on Gaussian charge carrier distribution with full-width at half maximum (FWHM) comprised between 1 and 3 µm, representative of the charge carrier distribution between *t* = 0 ns and *t* = 100 ns. Notably, its value is 1.5 µm when the fitting procedure starts at *t* = 10ns. The recycling term is represented in Supplementary Figure 2-a as a function of the radius *r*, along with the local PL emission intensity. At first sight, they behave similarly at short distances, but can strongly differ for large values of *r*. Supplementary Figure 2-b shows the local correction factor g_{corr}, as introduced in the model part, which is written in different terms here under:

$$g_{\text{corr}}(r) = 1 - \frac{g_{\text{rec}}(r)}{\iint n_\gamma(r, E, \Omega) dE d\Omega} = 1 - \frac{\text{recycling}}{\text{emission}} \quad (11)$$

For each value of FWHM, a threshold appears where the PL emission drops, whereas the PL recycling maintains its intensity, as the PL signal emitted at the center propagates and gets reabsorbed farther inside the material. As a consequence, the ratio $I_{\text{PL}}/g_{\text{rec}}$ drops drastically and g_{corr} values become negative (the recycling contribution becoming higher than the emission contribution). Past this threshold, the recycling is not proportional to the local emission anymore and our fitting approach is impossible. Nevertheless, this threshold is

above 3µm as soon as FWHM > 1.5µm. In other terms, the local correction factor g_{corr} remains constant around 0.43 ± 0.05 for 0 µm < r < 3 µm as soon as FWHM > 1.5 µm, which corresponds to our fitting window. Henceforth, our fitting approach is valid for t > 10 ns, when the charge carrier concentration is not too peaked.

In Supplementary Figure 3, the individual contributions of each wavelength to the recycling term are displayed. It shows that Supplementary Equations (8-9) (average absorption coefficient) are valid at short distances, but underestimate the long-range photonic transport. We tackle this long-range transport in the next section by considering a multi-wavelength PL flux.

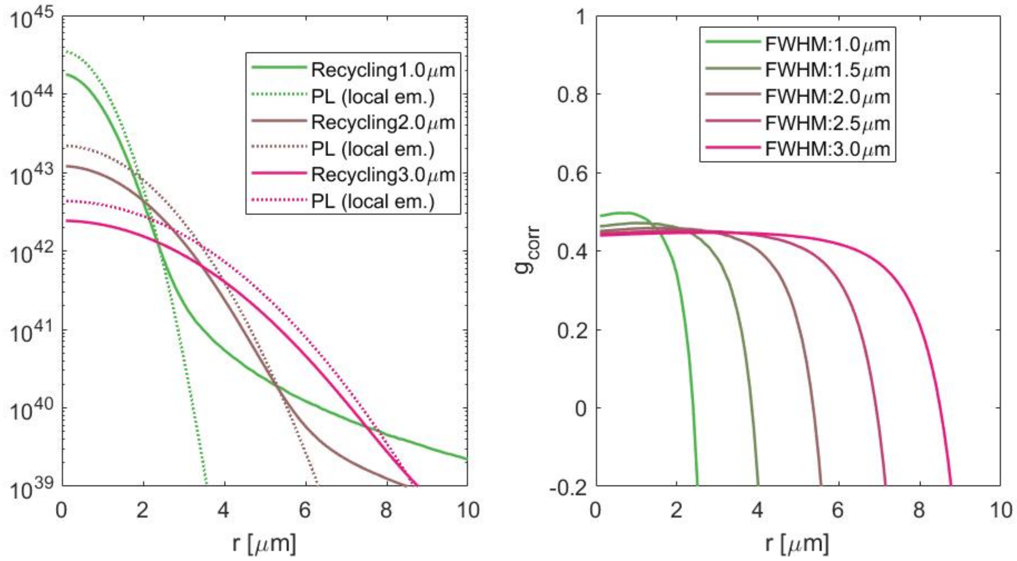

Supplementary Figure 2 (a) Contributions to rate equation (3) for a gaussian charge carrier distribution with FWHM=1..3µm, and $\Phi_0 = 5 \times 10^6$photons per pulse. Polychromatic PL is considered (no α_{PR}). The PL emission is displayed in dotted lines, while the recycling is in plain lines. Vertical scale is in arbitrary units. (b) The local R_{eh} correction factor, as defined in Supplementary Equation (11) is displayed for gaussian charge carrier distributions with FWHM=1..3μm.

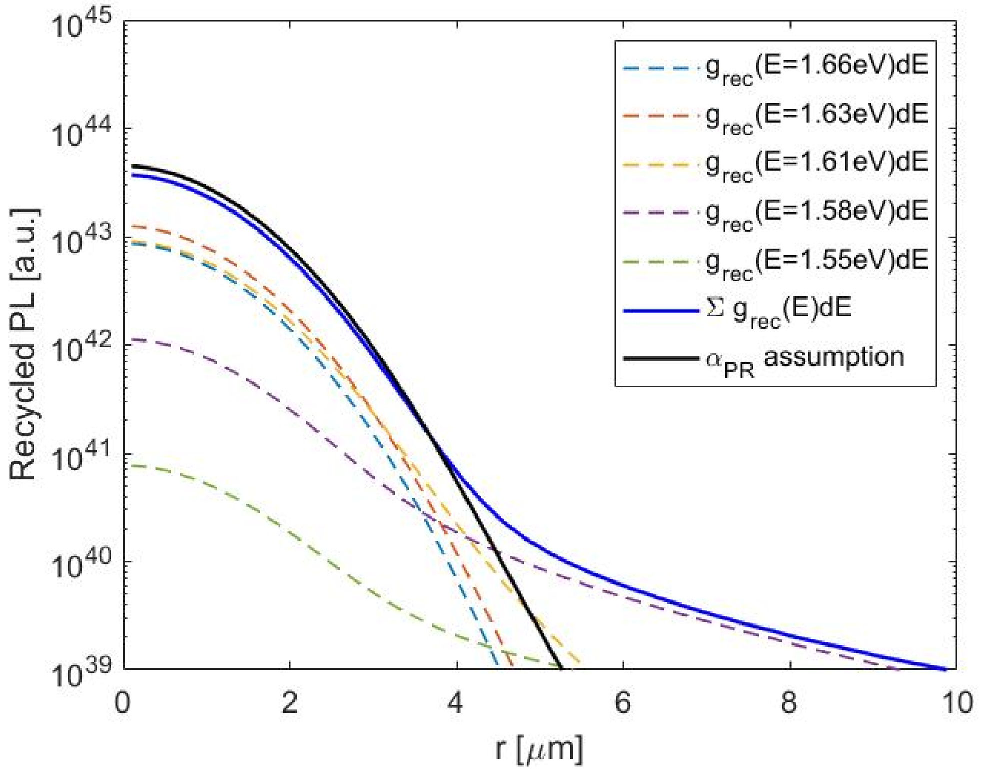

Supplementary Figure 3 Contribution of each PL wavelength to the recycling. The blue curve gives the sum of all contributions (Supplementary Equation (7)), and matches the red curve in Supplementary Figure 2-A. The black curve was derived under the assumption of a fixed absorption coefficient for the whole PL emission, as defined in Supplementary Equation (10).

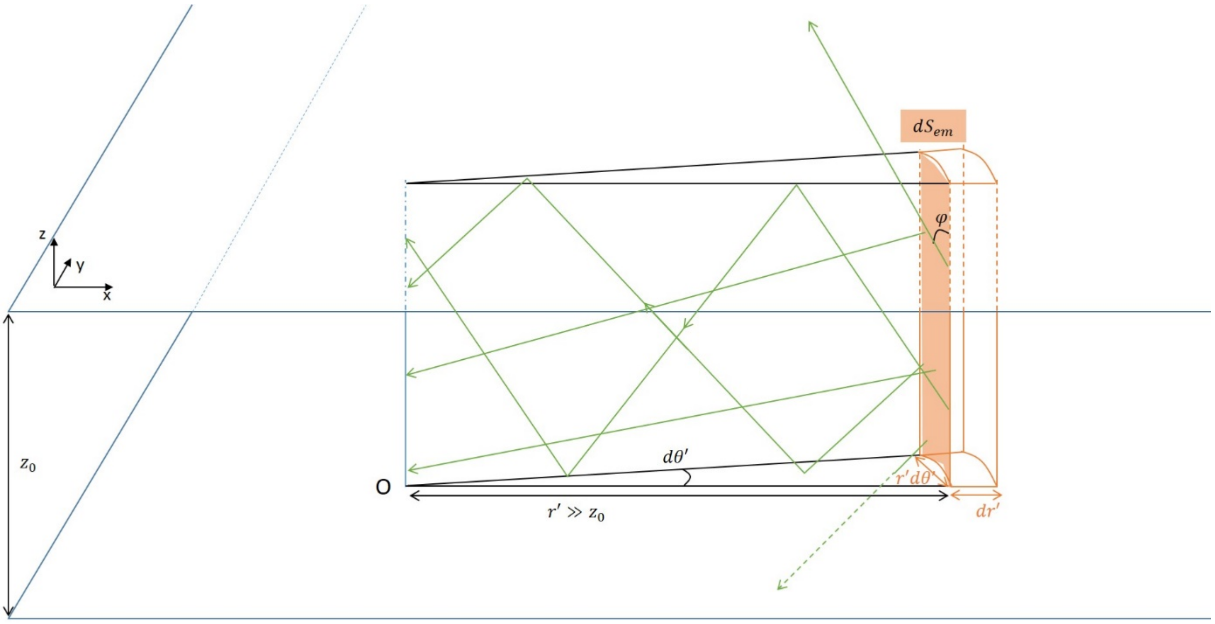

Supplementary Figure 4 Geometrical sketch showing the photons (green rays) emitted in the elementary volume in orange, towards the origin of a secondary frame, where the recycling term g_{rec} is calculated. Various solid angles of emission ultimately propagate towards the origin thanks to total internal reflection at front and back interfaces for $\varphi_{\mathrm{c}} < \varphi < \pi - \varphi_{\mathrm{c}}$.

Supplementary Note 3 Deriving propagated ϕ_{prop} and recycled g_{rec} contributions at longer range (r>>z0)

In this case, the infinitesimal volume becomes $dV = dr' \times r'd\theta' \times z_0$ (see Supplementary Figure 4) and the emission surface is $\mathrm{d}S_{\mathrm{em}} = r'd\theta' \times z_0$. The absorptivity of dV remains the same with $a(E) = \alpha(E)dr$. Total internal reflexions of the PL have to be considered and several photon trajectories link two points, as indicated with green rays in Supplementary Figure 4. We consider them by varying the angle φ between the PL ray and (Oz). The critical angle can be defined using Snell-Descartes law : $\varphi_{\mathrm{c}} = \mathrm{asin}(1/n_{\mathrm{opt}})$. For $\varphi < \varphi_{\mathrm{c}}$ and for $\varphi > \pi - \varphi_{\mathrm{c}}$, photons are emitted out of the thin film. For $\varphi_{\mathrm{c}} < \varphi < \pi - \varphi_{\mathrm{c}}$, they eventually reach the origin. To account for the Lambertian emission through the elementary surface dS_{em}, a solid angle of emission $d\Omega_{\mathrm{em}}(\varphi)$ has be defined. It reads:

$$d\Omega_{\mathrm{em}}(\varphi) = \frac{dS_O}{\frac{r'^2}{\sin^2\varphi}} = \frac{dS_O \sin^2\varphi}{r'^2} \quad (12)$$

The photon flux density at the origin $dn_{\gamma,2}$, having been emitted from the surface dS_{em} can be expressed as:

$$dn_{\gamma,2}(r',\theta',E) = \frac{K_{\mathrm{p}} n^2(r',\theta')}{n_0 p_0} \alpha(E) dr' E^2 \exp\left(-\frac{E}{k_{\mathrm{B}}T}\right) \int_{\varphi_{\mathrm{c}}}^{\pi-\varphi_{\mathrm{c}}} dS_{\mathrm{em}} \sin\varphi \, d\Omega_{\mathrm{em}}(\varphi) \exp\left(-\frac{\alpha(E)r}{\sin(\varphi)}\right) d\varphi \quad (13)$$

Then, for a charge carrier distribution located away from the origin, one can integrate $dn_{\gamma,2}$ over the semi-conductor slab to obtain the long-range propagated photon density per energy:

$$n_{\gamma,2}(E) z_0 dS_O = \frac{K_{\mathrm{p}}}{n_0 p_0} E^2 \alpha(E) \exp\left(-\frac{E}{k_B T}\right) \int_{r'=0}^{\infty} \int_{\theta'=0}^{2\pi} \int_{\varphi_{\mathrm{c}}}^{\pi-\varphi_{\mathrm{c}}} \frac{n^2(r',\theta') dS_O \, z_0 \sin^3\varphi}{r'} \exp\left(-\frac{\alpha(E)r'}{\sin(\varphi)}\right) dr' d\theta' d\varphi \quad (14)$$

Where the volume on which we determined the propagation can now be simplified:

$$n_{\gamma,2} = \frac{K_{\mathrm{p}}}{n_0 p_0} E^2 \alpha(E) \exp\left(-\frac{E}{k_{\mathrm{B}} T}\right) \int_{r'=0}^{\infty} \int_{\theta'=0}^{2\pi} \int_{\varphi_{\mathrm{c}}}^{\pi-\varphi_{\mathrm{c}}} \frac{n^2(r',\theta') \sin^3\varphi}{r'} \exp\left(-\frac{\alpha(E)r'}{\sin(\varphi)}\right) dr' d\theta' d\varphi \quad (15)$$

To determine the propagated flux at a certain distance *r* from the symmetry center of the charge carrier concentration, one needs to define a secondary frame with polar coordinates centered at the point (*r*,0), and then it is possible de calculate $\phi_{\mathrm{prop}}(r)$ within this secondary frame using Supplementary Equation (16).

$$\phi_{\mathrm{prop}}(r,E) = \iint_{(r',\theta')\ \text{frame centered in}\ (r,0)} \frac{K_{\mathrm{p}} E^2 \alpha(E) \exp\left(-\frac{E}{k_{\mathrm{B}}T}\right)}{n_0 p_0} \int_{\varphi_{\mathrm{c}}}^{\pi-\varphi_{\mathrm{c}}} \frac{n^2(r',\theta') \sin^3\varphi}{r'} \exp\left(-\frac{\alpha(E) r'}{sin\,\varphi}\right) dr' d\theta' d\varphi \quad (16)$$

We changed *r* (from the primary frame) from 3 to 20 µm and simulated the evolution of the spectrum and the intensity of the propagated PL, thereby generating a family of propagated spectra ϕ_{prop}. This model was verified by fitting the PL spectra extracted from hyperspectral maps acquired around a pointd pulsed illumination. They were reproduced with enhanced precision as the linear combination of the calculated propagated flux to a direct PL emission, taken as proportional to $I_{\mathrm{PL}}(r=0)$, described by Supplementary Equation (17).

$$\frac{I_{\mathrm{PL}}(r)}{\max_{\mathrm{E}}\left(I_{\mathrm{PL}}(r)\right)} = \beta \frac{\phi_{\mathrm{prop}}(r)}{\max_{\mathrm{E}} \phi_{\mathrm{prop}}(r)} + (1-\beta)\frac{I_{\mathrm{PL}}(r=0)}{\max_{\mathrm{E}} I_{\mathrm{PL}}(r=0)} \quad (17)$$

Normalizations are employed so that the sum of coefficients for both contributions is 1. Results are displayed in Supplementary Figure 5, and showcase an excellent reproduction quality. The relative weight of direct and waveguided contribution is indicated in the legend of each subplot. The weight of direct emission is 100% at 3 µm and decreases to 40% at r=9 µm. The weight of propagated spectrum follows an opposite trend.

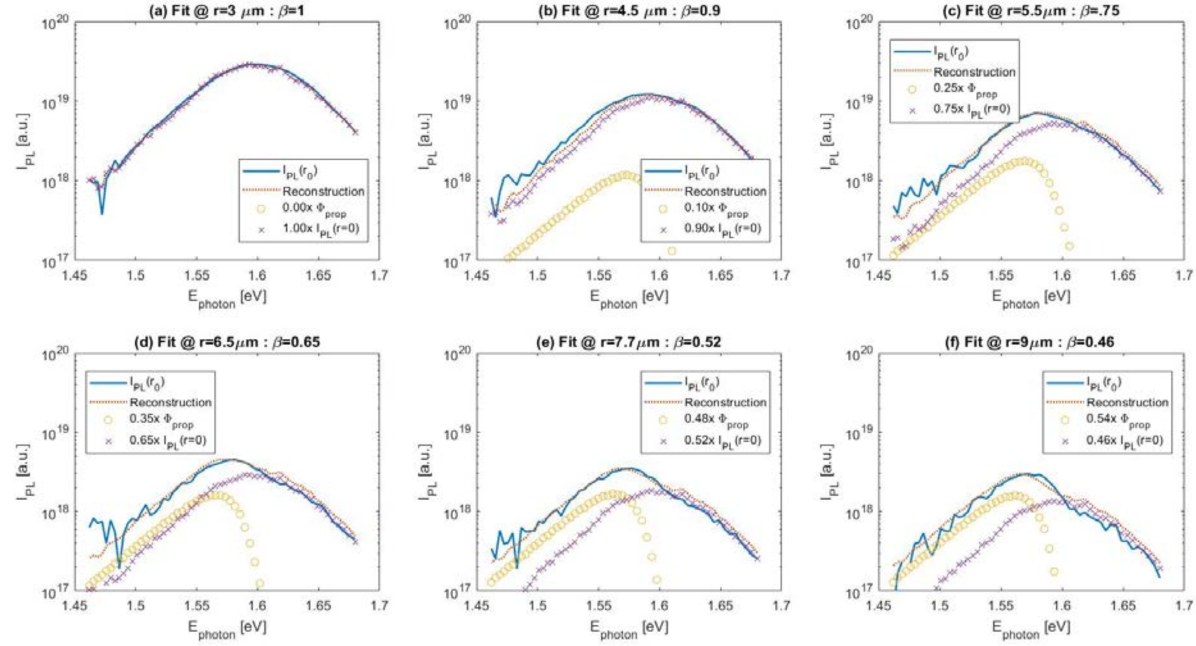

Supplementary Figure 5 Fitted reconstruction (red dotted line) of I_{PL} signal (blue line) for increasing distances to the excitation point. Fit is based on a linear combination of direct contribution (red crosses) and propagated one (yellow circle). The relative weight of the propagated contribution $(1-\beta)$ increases from 10 % at r = 4 µm to 54% at r = 9µm.

Supplementary Note 4 Insights into the fit of time-resolved diffusion profiles

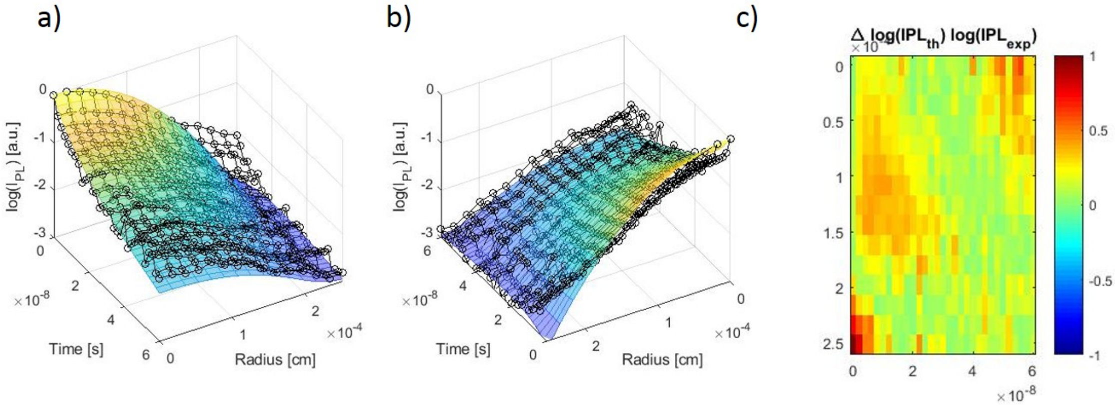

Supplementary Figure 6 Fitting the $I_{PL}(r,t)$ surface obtained at high fluence without the time domain restriction → Same diffusion coefficient estimated (0.024 ± 0.008 cm^2s^{-1} instead of 0.025 ± 0.006 cm^2s^{-1}) and τ_n=90 ± 50 ns instead of 110 ± 30 ns. R_{eh}* is evaluated at 6.7 ± 1 x 10^{-11} cm^3s^{-1}. This fit suffers from large values of the residuals, which rises at short times, and induces a worse quality of reproduction despite similar values fitted.

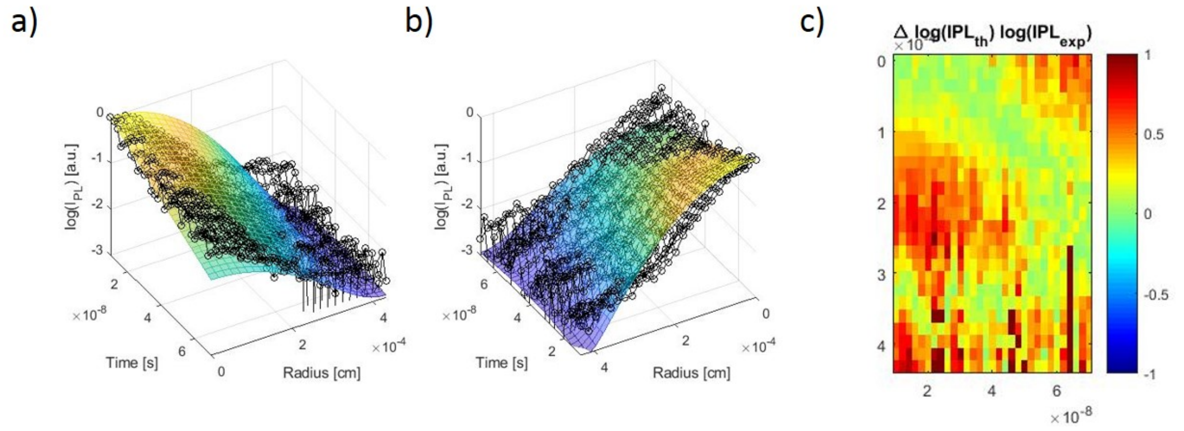

Supplementary Figure 7 Fitting the $I_{PL}(r,t)$ surface obtained at high fluence without the space domain restriction → Total wrong aspect + Diffusion coefficient over-estimated (0.1 ± 0.02 cm^2s^{-1} instead of 0.025 ± 0.006 cm^2s^{-1}) and τ_n= 50 ± 5ns instead of 70 ± 10ns. No precise estimation of R_{eh}^*…

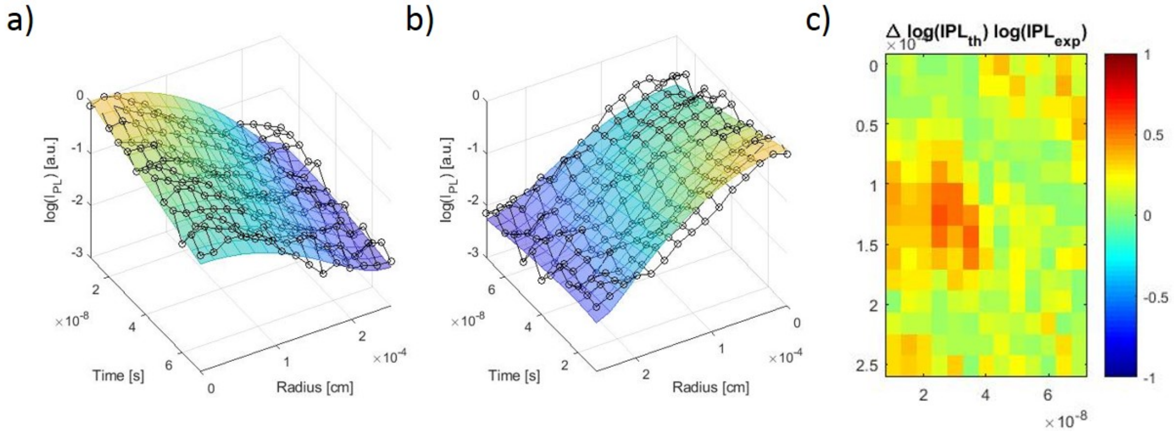

Supplementary Figure 8 Fitting the $I_{PL}(r,t)$ surface obtained at low fluence, with time and space-domain restriction yields an estimation of the SRH lifetime τ_n with 87 ± 30 ns instead of 110 ± 30 ns. Similar value of D_n with 0.037 ± 0.02 cm^2s^{-1} instead of 0.026 ± 0.007 cm^2s^{-1}. No precise estimation of $R_{eh,}^*$ as similar surfaces are obtained for 10^{-12} cm^3s^{-1} and for $R_{eh}^*=10^{-10}cm^3s^{-1}$, due to the low injection level.

Supplementary Note 5 Auger recombination influence

Auger recombination is a tri-molecular process that could contribute to the continuity equation once the charge carrier concentration is above 10^{18} cm^{-3} [4,5]. In our study, fits realized at low density are not impacted. For fits realized at high density, the concentration of charge carriers after 10 ns, when the fitting procedure begins, valuates at $4 \times 10^{17}/cm^3$ (10^{18} $/cm^3$ at t = 0 ns ; 10^{17} cm^{-3} at t = 50 ns), according to simulations realized with the parameters fitted in our study. To ensure that Auger influence does not disturb our kinetics analysis significantly, fits at high density were realized again with coefficients in the order of magnitude described by the literature. This is displayed in Supplementary Figure 9, where the title of each subplot indicates the value determined for D_n, R_{eh}^* and τ_n. We conclude that Auger recombination have a very small impact on the determined coefficients, especially the SRH lifetime and the diffusion coefficient which remain constant. If higher fluence were used, a more complete version of the continuity equation should be used to fit the results.

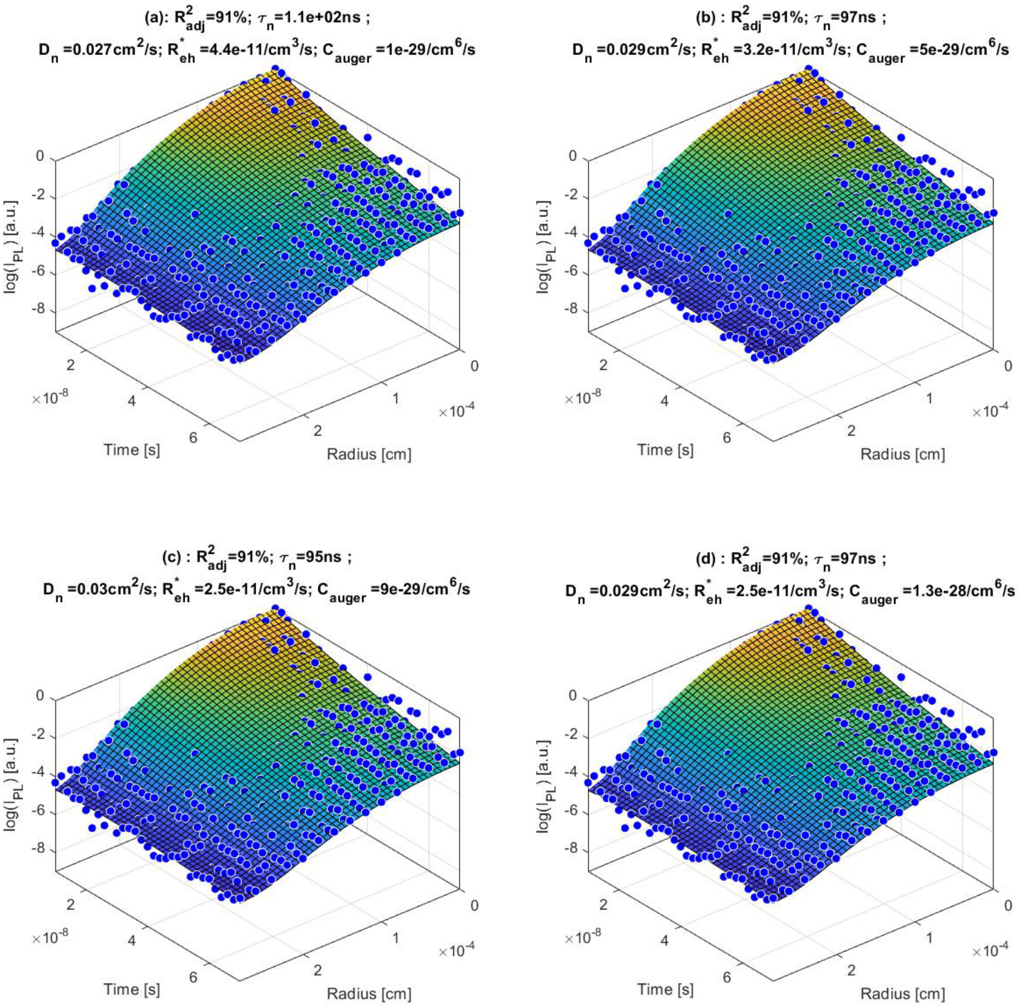

Supplementary Figure 9 Fitting the $I_{PL}(r,t)$ surface obtained at high fluence with the space and time-domain restriction, and accounting for Auger recombination. Various Auger coefficients C_{auger} are taken [a:10^{-29} ; b:5x10^{-29} ; c:9x10^{-29} ; d:1.3x10^{-28} cm^6 s^{-1}] in the range of literature values [6,7]. The impact on lifetime and diffusion coefficients values determined is negligible. The external radiative coefficient determined varies from 4.5 (a) to 2.5 (d) x$10^{-11}$$cm^3$/s.

Supplementary Note 6 Absorption properties of perovskite absorber

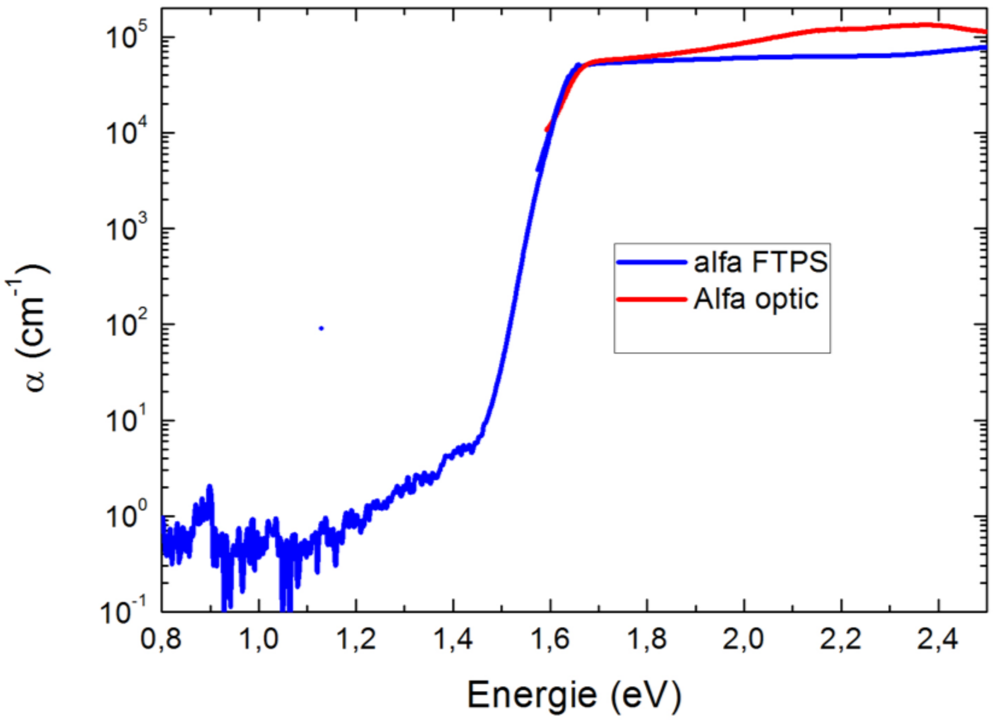

Supplementary Figure 10 Absorption coefficient of the investigated perovskite layer. Optical measurements are realized for *E* > 1.6eV, and serve for absolute calibration of FTPS (Fourier Transform Photocurrent Spectroscopy) measurements, which values should be considered for *E* > 1.6eV.

Supplementary Note 7 Illumination power not influencing the spectral shape of the PL

We realized this power study with a green continuous-wave laser illuminating a triple cation perovskite sample deposited on glass. Hyperspectral images were acquired and a mean spectrum was calculated on the illuminated zone ($\approx$ 60 μm^2). The spectra displayed in Supplementary Figure 11 do not shift as the injection is reduced. This appears even more clearly when they are normalized. Henceforth, power-induced effect cannot explain the spectral shift observed in Figure 2-AB.

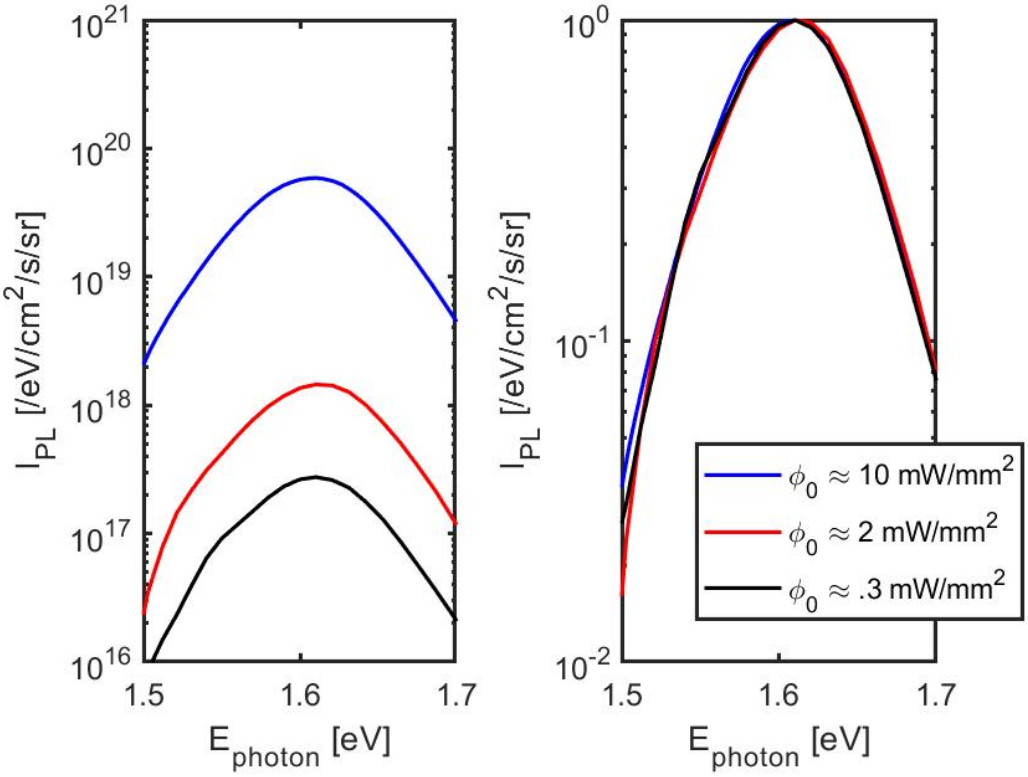

Supplementary Figure 11 (left) PL spectra obtained for increasing fluence with an illuminated surface of approx. 60µm² with a green continuous wave laser, represented in absolute units. (right) Normalized spectra underlining the stability of its shape with power

Supplementary References

1. Ramos, F. J. *et al.* Highly efficient MoOx-free semitransparent perovskite cell for 4 T tandem application improving the efficiency of commercially-available Al-BSF silicon. *Sci. Rep.* **8**, 16139 (2018).
2. van Roosbroeck, W. & Shockley, W. Photon-Radiative Recombination of Electrons and Holes in Germanium. *Phys. Rev.* **94**, 1558–1560 (1954).
3. Ahrenkiel, R. K. *et al.* Ultralong minority-carrier lifetime epitaxial GaAs by photon recycling. *Appl. Phys. Lett.* **55**, 1088–1090 (1989).
4. Ansari-Rad, M. & Bisquert, J. Insight into Photon Recycling in Perovskite Semiconductors from the Concept of Photon Diffusion. *Phys. Rev. Appl.* **10**, 034062 (2018).
5. Braly, I. L. *et al.* Hybrid perovskite films approaching the radiative limit with over 90 % photoluminescence quantum efficiency. *Nature Photonics.* ***12***, 355-361 (2018).
6. Huang, J., Yuan, Y., Shao, Y. & Yan, Y. Understanding the physical properties of hybrid perovskites for photovoltaic applications. *Nat. Rev. Mater.* **2**, (2017).
7. Staub, F., Kirchartz, T., Bittkau, K. & Rau, U. Manipulating the Net Radiative Recombination Rate in Lead Halide Perovskite Films by Modification of Light Outcoupling. *J. Phys. Chem. Lett.* **8**, 5084–5090 (2017).
